# Heterogeneity in family dynamics among adolescents engaging in high-risk sexual behaviors: a latent class analysis

**DOI:** 10.3389/fpubh.2025.1602085

**Published:** 2025-08-01

**Authors:** Sarah M. Godoy, Mimi V. Chapman, Todd M. Jensen, Eraka P. Bath, Laura S. Abrams, Rachel W. Goode, William J. Hall

**Affiliations:** ^1^Silver School of Social Work, New York University, New York, NY, United States; ^2^School of Social Work, University of North Carolina Chapel Hill, Chapel Hill, NC, United States; ^3^School of Education, University of North Carolina Chapel Hill, Chapel Hill, NC, United States; ^4^Division of Child and Adolescent Psychiatry, David Geffen School of Medicine, University of California, Los Angeles, Los Angeles, CA, United States; ^5^Department of Social Welfare, Luskin School of Public Affairs, University of California, Los Angeles, Los Angeles, CA, United States

**Keywords:** commercial sexual exploitation, child sex trafficking, sexually transmitted infections, child wellbeing, human trafficking, adolescent health, family characteristics, sexual risk taking behaviors

## Abstract

**Background:**

Little is known about whether and to what extent family factors associated with risky sexual behaviors, such as experiencing commercial sexual exploitation (CSE) or having prior sexually transmitted infections (STIs), differ across risk groups of adolescents.

**Methods:**

We conducted secondary analysis of a nationally representative dataset. Latent class analysis was used to assess heterogeneity in family characteristics and childhood adversity within classes of 1,018 adolescents who engaged in risky sexual behaviors, as evidenced by a self-reported STI and/or involvement in CSE. Participants were on average 15.49 years old (SD = 1.34), 50% female, and 58% people of color.

**Results:**

A five-class solution was deemed optimal. These classes were labeled: *abused/neglected/unloved* (12%); *disengaged dad/connected mom* (16%); *disengaged mom/minimally present dad* (9%); *connected and active parents* (35%); and *hiding in plain sight* class (15%). Adolescents who were CSE-impacted represented 49% of the analytic sample and were observed across all five classes in differing yet not insignificant proportions (i.e., ranging from 37% to 60%). Findings illustrate significant variability in family patterns and differences marked by demographic and sexual risk characteristics.

**Findings:**

The presence of CSE-impacted adolescents across latent classes speaks to the hidden nature of this crime and complexities related to CSE risk. Family relationships are often assumed to be protective against CSE. However, these findings point to considerable complexity in understanding how family functioning relates to CSE. Research that allows for longitudinal or retrospective analysis to understand how families were functioning at the time of CSE initiation, would help in delineating what types of families are most protective against CSE for adolescents.

## 1 Introduction

Commercial sexual exploitation (CSE), often used interchangeably with the term *sex trafficking*, is a largely hidden and complex social issue that disproportionately impacts structurally vulnerable adolescents (1, 2). For clarity and consistency, the term ‘adolescent’ is used throughout this article to refer broadly to individuals between ages 10 and 24, and is intended to encompass those otherwise described in the literature or data as children, youth, or young adults. Indeed, studies indicate that adolescents who identify as Black, Indigenous, and other people of color (BIPOC) and/or as lesbian, gay, bisexual, transgender, queer or questioning, and more (LGBTQ+) are vulnerable to CSE (1, 3). Further, those who face abuse, maltreatment, poverty, or material need are all at increased risk of CSE (1, 4). Research also suggests that engaging in high-risk sexual activities (e.g., unprotected sex, having a high number of sexual partners, having older sexual partners) and prior sexually transmitted infections (STIs) are indicative of CSE risk among adolescent boys and girls (5, 6). These vulnerabilities are magnified when adolescents are embedded in family systems characterized by caregiver strain or absence, poor nurturing, and conflict with parents (1, 2, 4, 6). In this regard, family dynamics are not only salient contributors to adolescents’ CSE pathways but may also affect access to support needed to successfully exit the exploitation (2).

Bronfenbrenner’s Bioecological Theory of Human Development provides a dynamic framework for understanding how adolescents’ vulnerability emerge from reciprocal interactions between individuals and their multilayered environments. The theory is defined by four interrelated components—Process, Person, Context, and Time—that influence one’s development across the lifespan (7, 8). Central to this theory are proximal processes, defined as sustained, reciprocal interactions between individuals and their environments, which drive development (7, 8). These interactions are shaped by personal characteristics, such as adolescents’ disposition, resources of ability (e.g., cognitive, emotional), and demand characteristics (e.g., behaviors that elicit responses from others), all of which can promote or hinder development (7). Context involves nested environmental systems ranging from immediate settings, such as family and school, to broader sociocultural structures, such as social norms (7). Time refers to the timing of life events and development transitions (micro-, meso-, and macro-time), which influence how processes and environments affect individuals over their lifespan (7). In the context of high-risk sexual behaviors, this theory highlights how family systems, personal vulnerabilities, and broader structural conditions converge and evolve over time to shape adolescents’ sexual risk behaviors and exposure to CSE.

Although CSE risk factors among adolescents are well-documented (1, 4, 6), less is known about the role of the family in the context of sexual risk-taking. For instance, very little research has compared differences in familial factors among (a) adolescents deemed high-risk for CSE due to their sexual risk-taking behavior and (b) those with confirmed CSE histories (5). Understanding differences in family characteristics and dynamics associated with risky sexual behavior may provide better insight into how families may effectively protect adolescents from or intervene when CSE is present.

Due to the complexities of identifying a largely hidden population, there are currently no available estimates of CSE in the United States (U. S.) (9). Nor are there universally accepted or codified definitions of CSE (2, 8). These logistical and conceptual challenges make it difficult to determine the scope of CSE or differences across adolescent’s experiences. These challenges are further complicated by the literature’s tendency to focus on CSE among women and adolescent girls (1, 10–12) and by media portrayals (e.g., images, news stories, television, movies) that sensationalize human trafficking (13, 14).

Contemporary discourse around human trafficking largely focuses on stereotypical notions of CSE. For instance, CSE is a perceived to be problem related to international borders or kidnapping by strangers. Simultaneously, media depictions predominately focus on the experiences of white, cis women and girls (1, 12, 13, 15). Together, research and media representations contribute to public perceptions of CSE rooted in stereotypes related to race/ethnicity, gender, and citizenship that do not account for the nuance and range of CSE experiences or the family contexts in which sexual risk-taking may occur. These stereotypes either elevate the risk of CSE or function as a warning sign that CSE occurs among particular groups (i.e., cis women and girls). Yet, CSE impacts many populations including cis boys, the LGBTQ+ community, and those of lower socioeconomic backgrounds. By examining sexual risk-taking behaviors among a wide range of adolescents, researchers can help identify family contexts and behaviors that may influence CSE entry.

### 1.1 Current study

To date, relevant review studies have consistently noted childhood adversity and dysfunctional family relationships as factors that contribute to CSE vulnerability (1, 6, 16). However, less is known about whether and to what extent family factors associated with risky sexual behaviors (e.g., childhood maltreatment) differ across risk groups. The primary objective of the present study was to examine heterogeneity in family characteristics and childhood adversity within classes of individuals who engaged in risky sexual behaviors, as evidenced by an STI diagnosis and/or experiences of CSE. To guide our understanding and interpretation of both shared and divergent experiences among adolescents, we drew upon Bronfenbrenner’s bioecological theory of human development, which emphasizes the dynamic interplay of individual characteristics and ecological contexts over time. The focal research questions were:

1. To what extent are there distinct subgroups marked by a unique set of family-related factors that were present among adolescents engaging in risky sexual behaviors? and.
2. Do these subgroups differ significantly by demographic or sexual risk characteristics? Findings may yield important implications for service providers’ understandings of CSE in the context of risky sexual behaviors and the role families can play in prevention and intervention strategies.

## 2 Materials and methods

### 2.1 Data and sample

Secondary data analysis was conducted using Waves I–IV of the National Longitudinal Study of Adolescent to Adult Health (Add Health) in-home interviews involving adolescents and parents. Add Health offers a nationally representative sample of 20,745 adolescents who were attending high school in the mid-90s in the U. S. (17, 18). Wave I data were collected among adolescent respondents between 1994 and 1995. Wave II data were collected approximately one-year later in 1996. Wave III data were collected from 2001 to 2002. Wave IV data were collected from 2008 to 2009. In-home parent surveys were collected at Wave I and included information from one parent about U. S. citizenship and family’s receipt of public assistance (e.g., welfare), among other factors (18). Add Health is one of the most comprehensive datasets capturing patterns of adolescent health, risk behavior, and family context, and is among the few available datasets that include measures relevant to CSE. As such, Add Health is uniquely suited to address the objectives of this study. Access to restricted data were granted by Carolina Population Center and approved by the University of North Carolina at Chapel Hill Institutional Review Board (IRB # 22–1,687).

The analytic sample was reduced to include adolescents who self-reported having a STI and/or experiencing CSE by Wave II. STI was measured by a variable that asked if adolescents had ever been told by a doctor or nurse that they had chlamydia, syphilis, gonorrhea, HIV or AIDS, genital herpes, genital warts, trichomoniasis, or hepatitis B. CSE was measured by a variable which asked if adolescents had exchanged sex for money or drugs. Only minor adolescents who had indicated “yes” to exchanging sex for money or drugs were included in this study as they are, by definition, considered sex trafficked or commercially sexually exploited [see (2)]. High-risk sexual behavior was operationalized using the variables: (1) STI; and (2) CSE. Thus, the current study was comprised of 1,018 adolescents engaged in high-risk sexual behaviors located across the U. S. Among the total sample, 502 adolescents were CSE-impacted and 516 reported a STI—a subset of adolescents experienced both CSE and a STI (*n* = 68). These adolescents were on average 15.49 years old (SD = 1.34; range: 12–17) at Wave I. About 50% of adolescents in the analytic sampled identified as female. In terms of racial/ethnic identity, approximately 58% identified as Black, Indigenous, Latinx, Asian/Pacific Islander, or American Indian and 42% identified as white. Of the respondents (*n* = 723) who disclosed their sexual identity at Wave IV, 79% identified as heterosexual and 21% identified as lesbian, gay, or bisexual (LGB).

### 2.2 Measures

#### 2.2.1 Family characteristics

Three items were used to assess family characteristics at Wave I. Family structure was measured as a nominal, categorical variable (1 = two biological parents; 8 = surrogate non-biological parent) (19). Responses were dichotomized (0 = no; 1 = yes) with a cut-off score of 5 to indicate if adolescents had or had not resided in a two-parent (i.e., biological, step, adoptive) household. Parents were asked to indicate (0 = no; 1 = yes) if they were a U. S. citizen and if they received public assistance.

#### 2.2.2 Child maltreatment

Two items were used to retrospectively assess childhood abuse at Wave III. Respondents were asked to indicate the level of frequency along a 6-point scale (1 = 1 time, 2 = 2 times, 3 = 3–5 times, 4 = 6–10 times, 5 = more than 10 times, and 6 = this has never happened to me) for the following items: (a) “How often had your parents or other adult care-givers slapped, hit, or kicked you?” and (b) “How often had one of your parents or other adult caregivers touched you in a sexual way, forced you to touch him or her in a sexual way, or forced you to have sexual relations?” Responses for these two items were dichotomized (0 = no; 1 = yes) with a cut-off score of 1 and combined into a single item to indicate if they had experienced any form of childhood abuse.

Two items were used to retrospectively assess childhood neglect at Wave III. Respondents were asked to indicate the level of frequency along a 6-point scale (1 = 1 time; 6 = this has never happened to me) for the following items: (a) “By the time you started 6th grade, how often had your parents or other adult care-givers left you home alone when an adult should have been with you?” and (b) “How often had your parents or other adult care-givers not taken care of your basic needs, such as keeping you clean or providing food or clothing?” Responses for these two items were dichotomized (0 = no; 1 = yes) with a cut-off score of 1 and combined into a single item to indicate if they had experienced any form of neglect.

At Wave IV, one item retrospectively measured feeling not wanted or loved in childhood. Respondents were asked to indicate the level of frequency along a 6-point scale (1 = 1 time; 6 = this has never happened to me) for the following item: “Before your 18th birthday, how often did a parent or other adult caregiver say things that really hurt your feelings or made you feel like you were not wanted or loved?” Responses were dichotomized (0 = no; 1 = yes) with a cut-off score of 1 to indicate whether they had experienced feeling unloved or unwanted in childhood. This item has been used to measure feeling not wanted or loved in other studies (20).

#### 2.2.3 Parental monitoring

Seven items were used to assess parental monitoring at Wave I. Adolescents were asked to indicate (0 = no; 1 = yes) if their parents let them make their own decisions about: (a) the time they must be home on weekend nights; (b) the people they hang around with; (c) what they wear; (d) how much television they watch; (e) which television programs they watch; (f) what time they go to bed on week nights; and (g) what they eat. Items were summed for a total score (0–7) for parental monitoring and dichotomized (0 = no; 1 = yes) with a cut-off score of 4 to indicate if adolescents had experienced lower levels of parental monitoring. These items have been used to measure parental monitoring in other studies (21).

#### 2.2.4 Parent–child interactions

A set of items, that have been successfully used in prior research (22, 23), indicated the quality of mother–child and father-child interactions, respectively, from the adolescents’ perspective at Wave I. Adolescents, including those in single- and two-parent households, were asked to indicate (0 = no; 1 = yes) if in the prior 4-weeks they had engaged in the following activities with their biological or residential mother figure or with their biological or residential father figure: (a) gone to a religious service or church-related event; (b) talked about someone they were dating or a party they went to; (c) had a talk about a personal problem they were having; (d) had a serious argument about their behavior; (e) talked about their schoolwork or grades; (f) worked on a project for school; and (g) talked about things they were doing in school. Adolescents that indicated that they had interacted on a particular item with their biological or residential mother/father figure scored 1 for that item. Two additional items were constructed to indicate if adolescents interacted with their mother or father, respectively, in any of the seven items previously listed (0 = no; 1 = yes). In total, 8 items were used to measure mother–child and father-child interactions, respectively.

#### 2.2.5 Family connections

Five items assessed mother–child and father-child relationship quality (24), respectively, at Wave I. Adolescents, including those in single- and two-parent households, were asked to indicate (a) how close they feel to their mother/father; (b) whether most of the time, their mother/father is warm and loving toward them; (c) their degree of satisfaction with the way their mother/father communicates with them; and (d) overall, how satisfied with their relationship with their mother/father. Item A was measured using a 5-point scale (1 = very little; 5 = very much) and for the remaining three items a 5-point scale (1 = strongly disagree; 5 = strongly agree). These items were coded such that higher values indicated higher levels of parent–child relationship quality. Responses were averaged and standardized for both mother–child relationship quality and father–child relationship quality. Cronbach’s alpha reliability was 0.81 for the items regarding mother–adolescent relationship quality and 0.71 for the items regarding father–adolescent relationship quality which indicates adequate internal consistency reliability.

Family belonging was measured using four items at Wave I. Adolescents, including those in single- and two-parent households, were asked to indicate their perceptions using a 5-point scale (1 = very little; 5 = very much): (a) “How much do you feel your family understands you?”; (b) “How much do you feel you want to leave home?” (reverse-coded); (c) “How much do you feel you and your family have fun together?” and (d) “To what extent do you feel your family pays attention to you?” These items were coded such that higher values indicated higher levels of family belonging. Responses were averaged and standardized. Cronbach’s alpha reliability was 0.74 for family belonging items which indicates adequate internal consistency reliability.

#### 2.2.6 Covariates

Analyses included covariates captured at Wave I, unless otherwise specified. The covariates were as follows: age group (ages 12–14 [0] or 16–17 [1]); gender (male [0] or female [1]); race/ethnicity (white [0] or BIPOC [1]); and sexual identity captured at Wave IV (heterosexual [0] or LGB [1]).

### 2.3 Analytic strategy

Spearman’s rank correlation coefficient, a non-parametric rank statistic, was used to examine associations between indicators (25). Latent class analysis (LCA) was used to identify the presence of unobserved subgroups of adolescents who have similar item-response patterns (23, 26, 27). LCA is a probabilistic modeling algorithm that allows for data clustering and statistical inference, and a form of mixture modeling that can account for categorical and continuous indicators (28). To minimize challenges related to the range of scales used in the observed indicators, all continuous variables were standardized (28). Meaning, continuous variables were placed on the same scale, specifically a z-scale, so that the mean was set to zero and the standard deviation to one (28).

In LCA, probabilities of class membership are estimated for each observation and used to assign adolescents to their most likely class (28, 29). Importantly, LCA is a person-oriented approach that assumes that a population is heterogeneous with respect to their relationship between variables and is used to describe similarities and differences *among the analytic sample* (30, 31). This is markedly different from a variable-centered approach that assumes that a population is homogenous and describes associations *among variables* that are believed to hold true for all individuals in a population (30, 31). This distinction is particularly important in the present study, as traditional variable-centered approaches may obscure subgroup patterns among adolescents engaging in high-risk sexual behaviors. Further, LCA addresses a gap in CSE literature, which has often treated family-related variables in isolation, rather than examining how they cluster together in meaningful ways. Thus, LCA uses multiple indicators to identify homogeneous subgroups in heterogenous populations (28). This analytic strategy enables researchers to explore adolescents’ experiences holistically and comprehensively (23, 26).

Model estimation was conducted by running a one-class model then added one latent class at a time (28). To identify the optimal latent-class solution, or class enumeration, indicators of model information, likelihood-based tests, indicators of classification uncertainty, and substantive interpretability were used (32–38). The model fit criteria and indices were: Akaike information criterion (AIC), Bayesian information criterion (BIC), and adjusted BIC (aBIC); bootstrap likelihood ratio tests (BS LRT); size of the smallest latent class; mean posterior probabilities; and entropy (23, 28, 39).

Information criteria, including AIC and BIC, are indices that seek to balance model complexity against the sample size, and aBIC is a sample-size adjusted form of BIC (23, 28, 39). Lower AIC, BIC, and aBIC values indicate a better fit (39). Likelihood-based tests, including BS LRT, compare relative fit of a model with *k* classes to a model with *k*-1 classes (23, 28). The BS LRT provide *p*-values to assess if adding a class leads to model fit improvement that is statistically significant (39). The smallest latent class size helps assess whether a latent class solution is over-extracted meaning that the model is extracting too many classes (23). Research suggests that class sizes should not have fewer than 50 cases or be at least 5% of the total sample (40).

Mean posterior probabilities indicate the average probability of class membership for individuals, conditional on item-response patterns, and range from values of 0 to 1 (23, 28, 39). Larger mean posterior probabilities, like 0.80 or higher, indicate greater accuracy in latent-class prediction (40). Entropy values are a measure of separation between latent classes and range from 0 to 1 (28). Entropy values approaching 1.0 indicate better class separation; therefore, values lower than 0.8 reflect poor class separation (41). Substantive knowledge and expertise related to CSE were integrated in determining the optimal number of classes (28). Taken together, these criteria and indices helped identify the optimal class solution. To address issues related to non-convergence and local loglikelihood maxima, which can result in anomalous findings, large random start sets were specified during analysis to find the global maximum (34, 42, 43).

The inclusion of covariates provides better insight into the heterogeneity of classes and enable us to assess construct validity of the latent-class solution (40, 44). Once the best-fitting solution, or optimal solution, was identified a three-step approach was used to estimate mean differences of covariates across classes (40, 45). Through this three-step approach classification uncertainty is accounted for by simultaneously extracting latent classes, computing true latent class membership, and assessing covariate mean-differences across classes (23, 45). Unweighted analyses were conducted in Mplus 8.4.

## 3 Results

### 3.1 Class enumeration and optional solution

Findings from Spearman’s correlations found indicators to be highly correlated (see Table 1). The class enumeration process for one-to-six classes is displayed in Table 2, including information about model fit criteria and indices. AIC, BIC, and aBIC values decreased as additional classes were extracted. The BS LRT *p*-value remained statistically significant from the 2-class to the 6-class solution, indicating improved model fit. The 5-class solution had the highest entropy value (0.84) and high mean posterior probabilities (range: 0.88–0.99). Based on these findings, coupled with substantive interpretation, a 5-class solution was deemed optimal and, therefore, retained. The 5-class solution revealed clear interpretation of five classes of adolescents with meaningful differences in family characteristics. In Table 3, the first column shows the overall means and response proportions across indicators for the full sample and subsequent columns show means and conditional response probabilities associated with each of the five extracted latent classes.

**Table 1:** Correlations between indicator variables.

	1	2	3	4	5	6	7	8	9	10	11	12	13	14	15	16	17	18	19	20	21	22	23	24	25	26
1. Two-parent household	1.00																									
2. Parent is U. S. citizen	−0.01	1.00																								
3. Received public assistance	−0.29***	0.02	1.00																							
4. Abuse	0.00	0.05	−0.08	1.00																						
5. Neglect	−0.10	0.06	−0.02	−0.40***	1.00																					
6. Felt unloved/unwanted	−0.08	0.07	−0.01	0.14	0.13	1.00																				
7. Parental monitoring (low)	−0.04	0.05	−0.06	−0.05	−0.07	−0.14	1.00																			
8. Attended religious service or event with mother	0.00	−0.03	−0.06	−0.02	−0.01	0.00	−0.10	1.00																		
9. Talked about dating or a party with mother	0.01	0.10	−0.10	−0.01	−0.01	0.03	0.13**	0.02	1.00																	
10. Talked about a personal problem with mother	−0.06	0.07	0.02	−0.04	0.06	0.06	0.07	0.07	0.35***	1.00																
11. Had serious argument with mother	−0.02	0.06	0.00	0.07	0.08	0.07	0.2	0.02	0.21***	0.21***	1.00															
12. Talked about school work or grades with mother	−0.07	0.07	0.01	−0.04	−0.02	0.01	0.02	0.15***	0.25***	0.23***	0.13**	1.00														
13. Worked on school project with mother	−0.02	0.01	0.01	−0.05	−0.08	0.03	−0.12	0.17***	0.04	0.12	0.07	0.24***	1.00													
14. Talked about other school things with mother	−0.05	−0.01	−0.01	0.00	−0.04	0.04	−0.01	0.13**	0.18***	0.18***	0.09	0.46***	0.27***	1.00												
15. No interactions with mother	−0.001	−0.05	0.02	0.01	−0.03	0.01	−0.05	−0.22***	−0.36***	−0.31***	−0.27***	−0.41***	−0.13***	−0.32***	1.00											
16. Attended religious service or event with father	0.05	−0.02	−0.10	−0.08	−0.01	−0.02	−0.11	0.52***	0.01	−0.04	−0.01	0.08	0.13	0.10	−0.10	1.00										
17. Talked about dating or a party with father	−0.02	0.09	−0.01	−0.05	−0.02	0.04	0.05	0.02	0.35***	0.20***	0.03	0.11	0.04	0.09	−0.10	0.11	1.00									
18. Talked about a personal problem with father	−0.10	0.04	0.06	−0.01	−0.04	0.07	0.00	−0.03	0.16**	0.32***	0.04	0.04	0.00	0.05	−0.10	0.08	0.39***	1.00								
19. Had serious argument with father	0.03	0.03	−0.07	0.01	0.02	0.05	0.06	−0.02	0.14*	0.11	0.28***	0.10	0.06	0.09	−0.06	0.08	0.13	0.13	1.00							
20. Talked about school work or grades with father	−0.11	0.03	0.02	−0.06	−0.03	0.04	−0.01	0.09	0.12	0.08	0.04	0.38***	0.18***	0.28***	−0.14*	0.13	0.24***	0.24***	0.16***	1.00						
21. Worked on a school project with father	−0.02	−0.01	−0.02	−0.01	0.02	0.06	−0.10	0.10	0.01	0.08	0.04	0.13*	0.28***	0.17***	−0.05	0.17**	0.l0	0.01	0.12	0.24***	1.00					
22. Talked about other school things with father	−0.09	0.02	−0.02	−0.04	−0.04	0.07	−0.01	0.11	0.07	0.07	0.02	0.26***	0.19***	0.43***	−0.16***	0.17***	0.26***	0.17***	0.10	0.48***	0.27***	1.00				
23. No interactions with father	−0.02	−0.07	0.06	0.04	0.00	−0.10	−0.03	−0.13	−0.13	−0.11	−0.03	−0.22***	−0.10	−0.18***	0.22***	−0.27***	−0.40***	−0.33***	−0.34***	−0.52***	−0.17***	−0.42***	1.00			
24. Mother/child quality relationship	0.05	0.00	−0.02	−0.09	−0.05	−0.09	−0.02	0.14**	0.04	0.07	−0.16***	0.13*	0.16***	0.13**	−0.05	0.10	0.01	−0.01	−0.08	0.01	0.06	0.05	0.01	1.00		
25. Father/child quality relationship	0.40***	0.03	−0.14**	0.02	−0.02	−0.11	0.00	0.06	0.01	−0.06	−0.09	−0.01	−0.02	0.01	−0.04	0.08	0.10	0.10	−0.02	0.05	0.02	0.09	−0.14*	0.13*	1.00	
26. Family belonging	0.03	−0.09	0.01	−0.09	−0.05	−0.14	−0.09	0.14**	0.00	0.01	−0.26***	0.15**	0.13*	0.13**	−0.05	0.09	0.03	0.05	−0.18***	0.08	0.05	0.10	−0.03	0.47***	0.25***	1.00

* indicates *p* ≤ 0.05. ** indicates *p* < 0.01. *** indicates *p* < 0.001.

**Table 2:** Model fit and class enumeration.

Classes	AIC	BIC	aBIC	BS LRT (*p*-value)	Entropy	Smallest *n*	Mean probabilities posterior					
							1	2	3	4	5	6
1	32478.02	32620.86	32528.75									
2	31142.03	31417.86	31240.00	0.00	0.76	388	0.92	0.93				
3	30571.82	30980.64	30717.03	0.00	0.78	217	0.92	0.90	0.88			
4	30152.71	30694.53	30345.16	0.00	0.82	133	0.89	0.89	0.92	0.90		
**5**	**29823.77**	**30498.57**	**30063.45**	**0.00**	**0.84**	**87**	**0.88**	**0.92**	**0.99**	**0.88**	**0.88**	
6	29637.26	30445.06	29924.18	0.00	0.82	87	0.89	0.90	0.86	0.84	0.83	0.99

AIC, Akaike information criterion; aBIC, adjusted Bayesian information criterion; aLRT, adjusted LRT; BIC, Bayesian information criterion; BS LRT, bootstrap LRT; LRT, likelihood ratio test. Bold items correspond with the optimal solution.

**Table 3:** Endorsement of family indicators in sample (*N* = 1,018) and across latent classes.

Indicator	Mean/proportion of “yes” responses	Latent class				
		1 (*n* = 125; 12%)	2 (*n* = 167; 16%)	3 (*n* = 87; 9%)	4 (*n* = 356; 35%)	5 (*n* = 283; 28%)
		Mean/conditional probability	Mean/conditional probability	Mean/conditional probability	Mean/conditional probability	Mean/conditional probability
Family Characteristics						
Two-parent household	0.62	0.49	**0.52**	**0.66**	**0.55**	**0.80**
Parent is U. S. citizen	0.88	**0.92**	**0.86**	**0.82**	**0.88**	**0.89**
Received public assistance	0.18	0.17	0.22	0.22	0.19	0.12
Childhood maltreatment and monitoring						
Abuse	0.34	**0.52**	0.38	0.33	0.28	0.31
Neglect	0.49	**0.64**	**0.54**	0.41	0.43	0.47
Felt unloved/unwanted	0.54	**0.77**	0.44	**0.53**	**0.57**	0.48
Parental monitoring (low)	0.68	**0.64**	**0.70**	**0.62**	**0.67**	**0.74**
Mother–Child Interaction						
Gone to a religious service or church-related event	0.32	0.20	0.26	0.00	0.46	0.32
Talked about someone you are dating or a party you went to	0.55	**0.54**	**0.53**	0.00	**0.68**	**0.57**
Had a talk about a personal problem you were having	0.48	0.41	0.48	0.00	**0.64**	0.47
Had a serious argument about your behavior	0.41	**0.70**	0.46	0.00	0.43	0.36
Talked about your school work or grades	0.61	**0.51**	**0.51**	0.00	**0.95**	0.48
Worked on a school project	0.13	0.06	0.09	0.00	0.31	0.02
Talked about other things you are doing in school	0.48	0.34	0.37	0.00	**0.89**	0.27
None	0.10	0.09	0.00	**1.00**	0.00	0.00
Father-Child Interaction						
Gone to a religious service or church-related event	0.22	0.19	0.00	0.07	0.33	0.26
Talked about someone you are dating or a party you went to	0.38	0.44	0.00	0.20	**0.54**	0.44
Had a talk about a personal problem you were having	0.30	0.39	0.00	0.13	0.41	0.34
Had a serious argument about your behavior	0.31	**0.52**	0.00	0.20	0.41	0.31
Talked about your school work or grades	0.51	**0.64**	0.00	0.27	**0.87**	0.41
Worked on a school project	0.10	0.14	0.00	0.05	0.23	0.01
Talked about other things you are doing in school	0.41	**0.50**	0.00	0.15	**0.79**	0.26
None	0.20	0.03	**1.00**	0.00	0.00	0.00
Family connection						
Mother/child quality relationship	0.00	−1.96	0.15	0.07	0.39	0.24
Father/child quality relationship	0.00	−0.35	−0.33	−0.08	0.02	0.34
Family belonging	0.00	−1.17	−0.05	−0.03	0.35	0.11

Conditional probabilities valued at 0.50 or higher are bolded to facilitate interpretation. Mother/child quality relationship (range: −3.75 — 0.99), father/child quality relationship (range: −2.24 — 1.63), and family belonging (range: −2.78 — 1.63) were standardized.

### 3.2 Latent class differences

Class 1 (*n* = 125; 12%), the *abused/neglected/unloved* class, had among the lowest probability of living in a two-parent household and the highest probability of parents being U. S. citizens. Across classes, these adolescents had the highest probabilities of experiencing abuse, neglect and feeling unloved and unwanted. This class was characterized as having low to moderate probabilities of interacting with mothers and fathers across the seven religious, social, or school-related items. Additionally, these adolescents had the highest probabilities of having a serious argument about their behavior with their mothers and fathers. The *abused/neglected/unloved* class had the lowest quality relationships with their mothers (*Z* = −1.96) and fathers (*Z* = −0.35), and the lowest sense of family belonging (*Z* = −1.17).

Adolescents in Class 2 (*n* = 167; 16%), the *disengaged dad/connected mom* class, were mostly living in a two-parent home with parents who were U. S. citizens. These adolescents were tied with Class 3 for the highest probability of their families receiving public assistance. Conditional probabilities indicate that about half of adolescents in this class experienced child neglect and had low parental monitoring yet had the lowest probability of feeling unloved and unwanted. All adolescents reported engaging with their mothers, at least to some degree, across the interaction items with high probabilities of discussing social life and schoolwork or grades. However, this class had no probability of engaging with fathers in any of the religious, social, and school-related interaction items. The *disengaged dad/connected mom* class had mixed parent–child quality relationships, specifically above average quality relationships with mothers (*Z* = 0.15) and below average quality relationships with fathers (*Z* = −0.33). Adolescents’ sense of family belonging was only slightly below average (*Z* = −0.05).

Adolescents in Class 3 (*n* = 87; 9%), the *disengaged mom/minimally present dad* class, was the least prevalent pattern. This class had a high probability of living in a two-parent home, the lowest probability of parents being U. S. citizens, and the highest probability of families receiving public assistance across classes. Though adolescents had the lowest probability of neglect, their conditional probabilities for abuse, neglect, and feeling unloved were still moderate to high. They also had the lowest probability of experiencing low parental monitoring, meaning they had less autonomy over decisions compared to other classes. This was the only class to have no probability of engaging with mothers in any of the seven religious, social, and school-related items. They also had low conditional probabilities of engaging in any of the seven activities with fathers. Still, adolescents in the *disengaged mom/minimally present dad* class had near-average relationship quality with mothers (*Z* = 0.07) and slightly below average with fathers (*Z* = −0.08) and their sense of family belonging was only slightly below average (*Z* = −0.03).

Class 4 (*n* = 356; 35%), the *connected and active parents’* class, was the most prevalent pattern. This class had the lowest probability of abuse and moderate to high probabilities of neglect, feeling unloved or unwanted, and low parental monitoring. Overall, these adolescents had highest conditional probabilities of engaging with mothers and fathers in all religious, social, and school-related items, except having a serious argument about their behavior. The *connected and active parents* class had the highest quality mother–child relationships (*Z* = 0.39) and sense of family belonging (*Z* = 0.35), and near average quality relationships with fathers (*Z* = 0.02).

Class 5 (*n* = 157; 15%), the *hiding in plain sight* class, had the highest conditional probability of residing in a two-parent household, a high probability that parents were U. S. citizens, and the lowest probability that their families received public assistance among classes. These adolescents had moderate probabilities of abuse, neglect, and feeling unloved or unwanted, and the highest probabilities of experiencing low parental monitoring, meaning they had more autonomy in decision-making than other classes. Overall, conditional probabilities of adolescents interacting with their mothers or fathers across the religious, social, and school-related items ranged widely from high to low. Adolescents had the highest probability of engaging in conversations about dating or a party with their mothers than interacting in other ways, and extremely low probabilities of working on school projects with mothers or fathers. These adolescents also had the second highest probabilities of attending a religious service or church-related event across classes. The *hiding in plain sight* class had the highest average for father-child quality relationship (*Z* = 0.34) among all classes, and above average quality relationships with mothers (*Z* = 0.24) and sense of family belonging (*Z* = 0.11).

### 3.3 Class differences by covariates

Differences in classes across covariates are displayed in Table 4. Adolescents who had CSE experiences, some of whom also had experienced an STI, represented 49% of the analytic sample and were the majority in the *disengaged mom/minimally present dad* class (60%) and *hiding in plain sight* class (55%). Those adolescents who self-reported a STI diagnosis (but no CSE history) comprised 51% of the analytic sample and were the majority in the *abused/neglected/unloved* class (63%) and *disengaged dad/connected mom* class (55%). The *connected and active parents* class had a near split of adolescents with a STI (51%) and the CSE group (49%). Adolescents ages 15–17 represented 77% of the analytic sample and were overrepresented in all five classes, relative to the sample proportion. Cis girls were 50% of the total sample and significantly overrepresented in the *abused/neglected/unloved* class (77%). The *disengaged dad/connected mom* class had only a slight majority of cis girls (52%) and the *connected and active parents* class had an even split of cis girls (50%) and cis boys (50%). Cis boys were overrepresented in *hiding in plain sight* (56%). Adolescents who identified as BIPOC comprised 58% of the analytic sample and were the majority in all classes. Lastly, adolescents who identified as LGB represented 21% of the analytic sample and had the highest proportion of adolescents in the *abused/neglected/unloved* class (34%).

**Table 4:** Latent classes differences by covariates.

Covariate	Full sample	1		2		3		4		5		Class differences, *p* ≤ 0.05
		(12%)		(16%)		(9%)		(35%)		(28%)		
	*N* = 1,018	*n* = 125		*n* = 167		*n* = 87		*n* = 356		*n* = 283		
	*M*	*M*	*SE*	*M*	*SE*	*M*	*SE*	*M*	*SE*	*M*	*SE*	
CSE	0.49	0.37	0.05	0.45	0.04	0.60	0.05	0.49	0.03	0.55	0.03	3 > 1, 2; 4 > 1; 5 > 1
Age group (15–17)	0.77	0.85	0.04	0.76	0.04	0.76	0.05	0.75	0.03	0.76	0.03	1 > 4, 5
Female	0.50	0.77	0.04	0.52	0.04	0.34	0.05	0.50	0.03	0.44	0.04	1 > 2, 3, 4, 5; 2 > 3; 4 > 3
BIPOC	0.58	0.58	0.05	0.58	0.04	0.61	0.05	0.63	0.03	0.52	0.04	4 > 5
LGB	0.21	0.34	0.06	0.17	0.04	0.20	0.05	0.20	0.03	0.19	0.03	1 > 2, 4, 5

Means and mean differences were estimated using the 3-step approach. Means represent class-specific proportions for binary/dummy variables. CSE, commercial sexual exploitation. BIPOC, Black, Indigenous, or other person of color. LGB, lesbian, gay, or bisexual.

## 4 Discussion

To our knowledge, this is the first study to use nationally representative data to identify heterogeneity in family characteristics and childhood adversity among sexually active adolescents who experienced CSE and/or a STI. Although this study investigated correlational links between latent classes and covariates, meaning that causal claims cannot be made based on our findings, these findings do illustrate significant variability in patterns of family characteristics and interactions as well as differences marked by demographic and sexual risk characteristics. As discussed, these findings alone have multiple implications for future practice and research.

Current literature suggests that poverty and maltreatment increase CSE risk, whereas living in a two-parent family structure, having a positive and supportive family, and feelings of family connectedness decrease CSE risk (6, 46–51). Yet, among adolescents already at risk, family factors that distinguish between CSE and other types of sexual risk are not as clear. Indeed, we observed CSE across all five classes in differing yet not insignificant proportions (i.e., classes ranged from 37 to 60%). While some adolescents faced moderate levels of childhood adversity stemming from their parental relationships, others who reported a greater sense of family belonging were still exploited. Some CSE-impacted adolescents were part of households receiving public assistance while others were not. These data indicate that it may be more difficult to detect CSE risk factors than previously assumed, particularly among adolescents already engaged in sex. Thus, future research should explore how different class profiles, such as adolescents with highly involved parents and high autonomy or those with disengaged parents, may require different screening protocols and intervention strategies.

Given that these data are cross-sectional and did not ask participants to self-report when the sex trading and/or STI occurred (e.g., age at onset), we do not know the timeline for these events. Therefore, understanding the connection with these high-risk sexual behaviors and parental relationships is complicated. To some degree, these findings call into question the extent to which family structures can protect against CSE once an adolescent is engaged in risky sex. To be included in the analytic sample, adolescents between the ages of 12 and 17 reported that they had already experienced at least one STI and/or had traded sex for money or drugs. LCA findings suggest that, within this already risk-exposed population, parental relationships matter.

Yet we observed that parental relationships alone might not have protected them from being sexually exploited, even when parents were highly engaged with their adolescents in religious, school, and social activities and topics. This may reflect a breakdown in proximal processes, or the regular, reciprocal interactions between adolescents and their environment that are essential for healthy development, as described in bioecological theory (8). For these interactions to be protective, they must occur consistently over time, become increasingly complex, and involve mutual responsiveness between the adolescent and others in their environment (8). When these conditions are not met, perhaps due to the interactions being superficial or inconsistent, the developmental benefits of proximal processes may be diminished (8). Another possible explanation is that for some of these adolescents both risky sexual behavior and/or CSE had stopped because family relationships had improved. Similarly, parents may have suspected or confirmed that sexual risk taking was occurring and therefore became more involved in their adolescent’s lives. Parental involvement may have included intervening, engaging in therapeutic services, and providing emotional support which together may have improved their overall relationships.

Alternatively, many adolescents may have hidden lives of which their parents are unaware. These adolescents may have had friends/peers or romantic partners who introduced them to and/or glamorized sexual risk-taking behaviors, despite being part of connected and engaged families (52). According to bioecological theory, interactions within peer microsystems, especially when these interactions are developmentally disruptive, can counteract the influence of supportive familial microsystems, particularly if the timing and intensity of peer influences overlap with critical development periods (8).

Our findings point to the need for interventions that go beyond general family engagement and include education on hidden risks, peer influence, and other grooming strategies, even when adolescents experience high levels of family connectedness. Prevention and intervention strategies may also target disengaged or minimally present caregivers with resources that support their involvement in their families, regardless of household structure (e.g., single-parent, two-parent households). This is consistent with bioecological theory’s emphasis on time (e.g., developmental stage, consistency of caregiver presence) and context (e.g., socioeconomic constraints, community supports), both of which shape how adolescents experience and respond to risk.

### 4.1 Debunking common stereotypes

CSE-impacted adolescents did not conform to many stereotypes highlighted in public discourse and associations documented in research. Findings from this study should dispel commonly held misconceptions about human trafficking that often oversimplify the issue in ways that diminish efforts to combat it. Many high-income countries perceive human trafficking as a prevailing issue among other countries. Indeed, media representations which suggest that exploitation is predominately an international issue or one that only affects foreign-born individuals trafficked into the U. S. are called into question by this research (13, 53, 54). Most adolescents in this study—and those in classes in which CSE-impacted adolescents were overrepresented—had high probabilities of having parents who were U. S. citizens. CSE must be understood as a domestic issue that affects adolescents and families born and embedded in our local communities. Despite the historically dominant public perception—both globally and locally—that adolescents are predominately kidnapped and lured from their homes into human trafficking (14, 54), we found that CSE-impacted adolescents had high probabilities of living in a two-parent household at the time of their exploitation. This finding aligns with existing literature indicating that adolescents may be trafficked while residing with their parents and through methods of entrapment that do not involve kidnapping or overt luring (52, 55). While not specifically studied in this analysis, some adolescents may be being exploited by parents or other family members (52, 56–58). The concerning disjunction between prominent narratives about CSE and these data further underlines the general lack of knowledge regarding this largely invisible crime.

Human trafficking narratives and discourse have predominately focused on the experiences of white, cis women and adolescent girls (10, 12, 52). Yet in classes in which we observed CSE was overrepresented (the *disengaged mom/minimally present dad* class and the *hiding in plain sight* class), the majority of adolescents identified as cis girls of color and white cis boys, with about 20% identifying as LGB. Thus, tailored interventions are needed for these populations, whose exploitation may not be obvious given their level of family connectedness. These findings align with literature suggesting that while girls of color, cis boys, and those in the LGBTQ+ community experience CSE, they remained under-studied in research and their under-identified as victims (10, 59, 60). Further, a notable proportion of adolescents in this study (24%) were exploited and/or had a STI by age 14. It is therefore plausible that sexual debut and CSE were initiated, at least for some, at much earlier life stages, which warrants further investigation of CSE entry. Exploring age of CSE onset is critical to challenging public misconceptions that adolescents are at greatest risk of sexual exploitation. More information about risk across early life stages can drive perceptions of and actions taken against human trafficking (56), and has important implications for how frontline professionals identify and respond to CSE across age groups.

### 4.2 Implications for practice

The presence of CSE-impacted adolescents across latent classes speaks to the hidden nature of this crime and complexities related to CSE risk. Once adolescents have engaged in sexual risk-taking behaviors, it may be even more difficult to detect CSE and further complicate caregivers’ abilities to protect their adolescents from sexual exploitation or intervene when it occurs. Likewise, healthcare providers (e.g., primary care providers, public health and community healthcare providers), who may be interacting with adolescents because of STIs or reproductive health needs, may assume that the sex adolescents are engaging in is consensual. Healthcare providers should further investigate the nature of adolescents’ sexual behaviors and if abuse or exploitation may be present. Further, given the severe under-investigation and potentially unique needs of cis boys and those in the LGBTQ+ community who experience CSE (10, 49, 59), it is crucial that service providers recognize that CSE-impacted adolescents are not a monolithic group. Indeed, participants in this study varied in racial/ethnic, gender, and sexual identities and included adolescents as young as 12 years old. Prevention curricula and trainings that integrate lived experience experts from diverse social groups and whose stories reflect a wide range of trafficking types and family contexts can build providers’ awareness of CSE forms and prevalence. These trainings should emphasize that exploitation can occur within seemingly stable families and among adolescents who appear supported or connected to their families. These approaches can help further dispel previously held misconceptions about whom CSE affects and how it occurs.

Some adolescents in this study may have been exploited by their parents or other family members (58, 61). Research suggests that children exploited by family members tend to be exploited at much younger ages and for longer periods of time than those exploited by non-relatives, and that they may feel pressure to maintain secrecy and protect familial traffickers from prosecution (57, 62). The resulting difficulty of identifying familial trafficking highlights the need for targeted screening tools that can assess for exploitation and distinguish among features of different trafficker types, including parents, romantic partners, and strangers. Given that adolescents in this study were attending middle and high school, school systems must build capacity for school nurses and social workers to assess students’ CSE risk. Further, it is critical that schools establish protocols to ensure that those who experience CSE are connected to specialized treatment and resources that support their immediate and long-term biopsychosocial needs. Lastly, schools should integrate CSE prevention programs into sexual health courses that proactively educate children about risk factors and protective strategies, including how to recognize grooming behaviors, build healthy relationships, and seek help from trusted adults.

More than half of adolescents in this study had been told by a doctor or nurse that they had a STI, of which about 12% were also experiencing CSE. This finding aligns with prior research that documents that at least some CSE-impacted adolescents engage in emergency and reproductive healthcare while experiencing exploitation (63–65). Thus, healthcare providers may serve an important role in CSE identification and should build capacity to screen for CSE risk. Additionally, adolescents in this study experienced moderate to high probabilities of abuse, neglect, and feeling unloved or unwanted, even in the presence of positive parent–child relationships, family belonging, and parent–child interactions. These findings underline the need for healthcare providers to intentionally screen for potential maltreatment in private rooms away from caregivers and in contexts when adolescents are being tested for or diagnosed with a STI. Indicators of maltreatment or that adolescents feel unloved and unwanted in their families should prompt CSE assessment and must be considered alongside other health outcomes, including those that are frequently overlooked (e.g., malnutrition, eating disorders), and social factors (e.g., pressure from peers or romantic relationships to engage in sex) that may suggest risk (1, 66).

Lastly, medical and public health professionals can partner with lived experience experts to help develop and implement school-, community- and hospital-based programs that provide relevant psychoeducation and incorporate skill building for adolescents and their families. Given that our findings highlight the presence and influence of parents, it is critical that guardians receive targeted training and resources related to CSE. These prevention strategies can help protect adolescents and equip guardians and caretakers with needed knowledge of how to detect CSE or potentially intervene. In addition, these programs should promote open communication within families about pressures to engage in sex, healthy relationships, and safe dating practices, which may further mitigate CSE risk.

### 4.3 Implications for research

This study provided greater insight into how adolescents engaging in sexual risk-taking differed across family patterns, and these insights have several implications for future research. While we observed significant associations between family dynamics and demographic/sexual risk characteristics, it was impossible to detect causality. Therefore, future research should use longitudinal designs to focus on detecting the causal nature of CSE and family dynamics by investigating familial factors that preceded CSE in addition to the potential moderating effects of sociodemographic characteristics. Future research can expand on this study by investigating how other potentially salient factors, such as domestic violence, community involvement, and religiosity, are correlated with family dynamics in the context of sexual risk taking and CSE.

Adolescents in this study had relatively low levels of perceived parental monitoring across classes, indicating higher levels of autonomy in decisions that affected their daily lives. Prior research has documented that lower levels of parental monitoring are associated with sexual risk behaviors among adolescents (67) while higher parental monitoring protects against sexual risk behaviors (68). Still, it is difficult to untangle the role of parental monitoring when adolescents are already engaging in sex and to determine exactly what “good” parental monitoring means for adolescents more generally. This study could not determine if there were certain aspects of parental monitoring at particular ages that can help guard against CSE. Future research should investigate what types of parental monitoring might serve a protective role and at what ages for adolescents who are at-risk or have confirmed experiences of CSE.

As noted, CSE research focusing on heterosexual boys is scant. We do not know enough about their experiences in any regard to have a reliable baseline understanding of their CSE pathways. In fact, this study indicates that positive family dynamics may be present even while heterosexual boys experience CSE. More research is needed to unpack if these adolescents’ experiences are anomalous or represent a particular profile of exploited adolescents.

Future research using qualitative or mixed method designs could provide a more in-depth understanding of how CSE interacts with family dynamics and social contexts across diverse social groups, including very young children, adolescents with disabilities, those who identify as LGBTQ+, and caregivers. Additional family characteristics not available in the Add Health dataset could be of particular interest. For instance, investigating the role of caregivers’ harmful family beliefs about women and children, religious beliefs and connectedness to religious or cultic groups, and mental health issues (including addiction) could provide greater insight into how and which family dynamics affect CSE pathways.

### 4.4 Strengths and limitations

The current study has several limitations, especially related to the nature of the variables available in the Add Health dataset, and findings should be interpreted with these in mind. The CSE measure was based on a single item that asked about trading sex for drugs or money. This definition of CSE is severely limited and does not capture instances of sex being traded for other items of value (e.g., food, shelter), the CSE experiences of adolescents who were unaware that anything of value was exchanged, or those who are unwilling to disclose sex trading. Thus, CSE may be underreported in Add Health. Further, information on adolescents’ CSE pathways was not available in Add Health. Therefore, it was impossible to determine if family dynamics were correlated with adolescents’ relationships with their traffickers, the age at which CSE was initiated, or the length of their exploitation, among other CSE-related factors. There were a host of other potential indicators and covariates that were not available in Add Health (e.g., issues with parental mental or physical health, family belief systems, parents’ physical absence due to work or other circumstances) that may have been correlated with family dynamics. A more holistic understanding of sexual exploitation among adolescents may be gained by investigating these additional CSE and family factors.

Our findings are also limited by the nature of the available sample. All adolescents in this study identified as cis boys or girls and were enrolled in school at Wave I. Therefore, findings are not representative all adolescents in this age group, especially those who identify as transgender, non-binary, or other gender expansive identities and/or were not enrolled in school.

Lastly, variables used in these analyses were self-reported and represent a static, cross-sectional view taken from Waves I–IV. For instance, adolescents were asked to report data on their interactions with mothers and fathers in the prior 4 weeks at Wave I. Therefore, these findings are not fully representative of adolescents’ interactions with their parents across the adolescent life stage, but rather provide a snapshot of interactions across a single month in adolescence. Moreover, data were collected between 1994 and 2009, which means that new trends and factors likely emerged in the years since, particularly in light of evolving societal trends such as the rise of technology and social media. The dated nature of the dataset should be considered when interpreting findings. Future research can examine these dynamics using more current, longitudinal data that capture contemporary influences on adolescent vulnerability as it relates to family dynamics and characteristics.

Despite these limitations, this study fills a critical knowledge gap in the human trafficking literature regarding aspects of familial dynamics associated with CSE. These findings point to critical differences in family characteristics, childhood adversity and monitoring, parent–child interactions, and family connection among adolescents affected by CSE and their sexual risk-taking counterparts. Understanding the pathways from family patterns to CSE can help guide community-based response efforts and future research that aims to eradicate child sexual exploitation.

## 5 Conclusion

This study provided insight into differences in family characteristics among sexually risk-taking adolescents at risk of or with confirmed experiences of CSE. Notably, while some adolescents reported moderate levels of childhood adversity and low levels of parent–child interactions, others described a stronger sense of family belonging and parental interactions and were still exploited. These findings challenge dominant assumptions that only adolescents who experience household dysfunctional or maltreatment are vulnerable to CSE. Both researchers and service providers must work together to debunk common human trafficking stereotypes that not only misrepresent the reality but are harmful to CSE-impacted individuals who do not fit within these dominant narratives. In particular, healthcare professionals in close proximity to adolescents must be equipped with comprehensive, trauma-informed training that goes beyond common stereotypes to recognize subtle signs of CSE. Expanding knowledge of the heterogeneity in family dynamics or other social contexts can help change public perceptions of CSE’s contexts and appearances, and ultimately drive public support for preventing and stopping the exploitation of children. Targeted educational initiatives for adolescents in schools, caregivers in community settings, and providers across settings can foster proactive engagement in prevention strategies and support the protection and recover of those at-risk of and impacted by CSE.

## Data Availability

Publicly available datasets were analyzed in this study. This data can be found: https://addhealth.cpc.unc.edu/data/.

## References

[1] Franchino-Olsen H. Vulnerabilities relevant for commercial sexual exploitation of children/domestic minor sex trafficking: a systematic review of risk factors. Trauma Violence Abuse. (2021) 22:99–111. doi: 10.1177/1524838018821956, PMID: 30712473

[2] Gerassi L. From exploitation to industry: definitions, risks, and consequences of domestic sexual exploitation and sex work among women and girls. J Hum Behav Soc Environ. (2015) 25:591–605. doi: 10.1080/10911359.2014.991055, PMID: 26726289 PMC4696486

[3] Williamson E Flood A. Systemic and structural roots of child sex trafficking: the role of gender, race, and sexual orientation in disproportionate victimization In: Chisolm-Straker C Chon K, editors. The historical roots of human trafficking. Cham: Springer (2021). 191–216.

[4] Choi KR. Risk factors for domestic minor sex trafficking in the United States: a literature review. J Forensic Nurs. (2015) 11:66–76. doi: 10.1097/JFN.0000000000000072, PMID: 25996431

[5] Hornor G Hollar J Landers T Sherfield J. Healthcare use and case characteristics of commercial sexual exploitation of children: teen victims versus high-risk teens. J Forensic Nurs. (2023) 19:160–9. doi: 10.1097/JFN.0000000000000402, PMID: 37590938

[6] Mercera G Kooijmans R Leijdesdorff S Heynen E van Amelsvoort T. Risk and protective factors for sexual exploitation in male and female youth from a cross-cultural perspective: a systematic review. Trauma Violence Abuse. (2023) 25:15248380231201815. doi: 10.1177/15248380231201815, PMID: 37818954

[7] Bronfenbrenner U Morris PA. The bioecological model of human development In: Lerner RM Damon W, editors. Handbook of child psychology: Theoretical models of human development. 6th ed. Hoboken, NJ: John Wiley & Sons (2006). 793–828.

[8] Tudge JR Mokrova I Hatfield BE Karnik RB. Uses and misuses of Bronfenbrenner’s bioecological theory of human development. J Fam Theory Rev. (2009) 1:198–210. doi: 10.1111/j.1756-2589.2009.00026.x

[9] Franchino-Olsen H Chesworth BR Boyle C Rizo CF Martin SL Jordan B. The prevalence of sex trafficking of children and adolescents in the United States: a scoping review. Trauma Violence Abuse. (2022) 23:182–95. doi: 10.1177/1524838020933873, PMID: 32588741 PMC8685723

[10] Allan C Winters GM Jeglic EL. Current trends in sex trafficking research. Curr Psychiatry Rep. (2023) 25:175–82. doi: 10.1007/s11920-023-01419-7, PMID: 37074570 PMC10113716

[11] Greenbaum VJ. Commercial sexual exploitation and sex trafficking of children in the United States. Curr Probl Pediatr Adolesc Health Care. (2014) 44:245–69. doi: 10.1016/j.cppeds.2014.07.001, PMID: 25131563

[12] Ottisova L Hemmings S Howard LM Zimmerman C Oram S. Prevalence and risk of violence and the mental, physical and sexual health problems associated with human trafficking: an updated systematic review. Epidemiol Psychiatr Sci. (2016) 25:317–41. doi: 10.1017/S2045796016000135, PMID: 27066701 PMC7137602

[13] Houston-Kolnik JD Soibatian C Shattell MM. Advocates’ experiences with media and the impact of media on human trafficking advocacy. J Interpers Violence. (2020) 35:1108–32. doi: 10.1177/0886260517692337, PMID: 29294657

[14] Rodríguez-López S. (De) constructing stereotypes: media representations, social perceptions, and legal responses to human trafficking. J Hum Trafficking. (2018) 4:61–72. doi: 10.1080/23322705.2018.1423447

[15] Khabbaz T. L. (2022). Exploring school personnel’s perceptions of a state sex trafficking policy. [dissertation]. Lehigh University;

[16] Puigvert L Duque E Merodio G Melgar P. A systematic review of family and social relationships: implications for sex trafficking recruitment and victimisation. Fam Relat Soc. (2021) 11:534–50. doi: 10.1332/204674321X16358719475186

[17] Harris KM. The Add Health study: Design and accomplishments. Chapel Hill, NC: Carolina Population Center, University of North Carolina at Chapel Hill. (2023). Available at: https://addhealth.cpc.unc.edu/wp-content/uploads/docs/user_guides/DesignPaperWave_I-IV.pdf

[18] Harris KM Halpern CT Whitsel EA Hussey JM Killeya-Jones LA Tabor J. Cohort profile: the national longitudinal study of adolescent to adult health (add health). Int J Epidemiol. (2019) 48:1415–1415k. doi: 10.1093/ije/dyz115, PMID: 31257425 PMC6857761

[19] Harris KM Ryan S. Father involvement and the diversity of family context In: Day RD Lamb ME, editors. Conceptualizing and measuring father involvement. Mahwah (NJ): Lawrence Erlbaum Associates (2004). 93–319.

[20] Ahuja M Okoro J Frimpong E Doshi RP Wani RJ. Feeling not wanted/loved and depression: does gender matter? Psychol Rep. (2023) 126:712–26. doi: 10.1177/00332941211062822, PMID: 34969338

[21] Harris-McKoy D. Adolescent delinquency: is too much or too little parental control a problem? J Child Fam Stud. (2016) 25:2079–88. doi: 10.1007/s10826-016-0383-z

[22] Ford CA Pool AC Kahn NF Jaccard J Halpern CT. Associations between mother-adolescent and father-adolescent relationships and young adult health. JAMA Netw Open. (2023) 6:e233944–4. doi: 10.1001/jamanetworkopen.2023.3944, PMID: 36943264 PMC10031392

[23] Jensen TM. Patterns of interactions between youth and resident stepmothers: a latent class analysis. Fam Process. (2021) 60:538–55. doi: 10.1111/famp.12556, PMID: 32648288

[24] Johnson MD Galambos NL. Paths to intimate relationship quality from parent–adolescent relations and mental health. J Marriage Fam. (2014) 76:145–60. doi: 10.1111/jomf.12074

[25] Hauke J Kossowski T. Comparison of values of Pearson's and spearman's correlation coefficients on the same sets of data. QUAGEO. (2011) 30:87–93. doi: 10.2478/v10117-011-0021-1

[26] Collins LM Lanza ST. Latent class and latent transition analysis: With applications in the social, behavioral, and health sciences. Hoboken: John Wiley & Sons (2010).

[27] Lanza ST Tan X Bray BC. Latent class analysis with distal outcomes: a flexible model-based approach. Struct Equ Modeling. (2013) 20:1–26. doi: 10.1080/10705511.2013.742377, PMID: 25419096 PMC4240499

[28] Sinha P Calfee CS Delucchi KL. Practitioner’s guide to latent class analysis: methodological considerations and common pitfalls. Crit Care Med. (2021) 49:e63–79. doi: 10.1097/CCM.0000000000004710, PMID: 33165028 PMC7746621

[29] Kainz K Jensen T Zimmerman S. Cultivating a research tool kit for social work doctoral education. J Soc Work Educ. (2018) 54:792–807. doi: 10.1080/10437797.2018.1434446

[30] Laursen B Hoff E. Person-centered and variable-centered approaches to longitudinal data. Merrill-Palmer Q. (2006) 52:377–89. doi: 10.1353/mpq.2006.0029

[31] Masyn KE. Latent class analysis and finite mixture modeling In: Little TD, editor. Oxford handbook of quantitative methods in psychology: vol. 2 Oxford, UK: Oxford University Press (2013). 551–611.

[32] Aflaki K Vigod S Ray JG. Part II: a step-by-step guide to latent class analysis. J Clin Epidemiol. (2023) 159:348–351. doi: 10.1016/j.jclinepi.2023.05.025 37286148

[33] Erosheva EA Matsueda RL Telesca D. Breaking bad: two decades of life-course data analysis in criminology, developmental psychology, and beyond. Annu Rev Stat Appl. (2014) 1:301–32. doi: 10.1146/annurev-statistics-022513-115701

[34] Jung T Wickrama KAS. An introduction to latent class growth analysis and growth mixture modeling. Soc Personal Psychol Compass. (2008) 2:302–17. doi: 10.1111/j.1751-9004.2007.00054.x

[35] Nagin DS Odgers CL. Group-based trajectory modeling in clinical research. Annu Rev Clin Psychol. (2010) 6:109–38. doi: 10.1146/annurev.clinpsy.121208.131413, PMID: 20192788

[36] Neely-Barnes S. Latent class models in social work. Soc Work Res. (2010) 34:114–21. doi: 10.1093/swr/34.2.114

[37] Nylund KL Asparouhov T Muthén BO. Deciding on the number of classes in latent class analysis and growth mixture modeling: a Monte Carlo simulation study. Struct Equ Modeling. (2007) 14:535–69. doi: 10.1080/10705510701575396

[38] Petras H Masyn K. General growth mixture analysis with antecedents and consequences of change In: Piquero AP Weisburd D, editors. Handbook of quantitative criminology. New York, NY: Springer (2010). 69–100.

[39] Nylund-Gibson K Choi AY. Ten frequently asked questions about latent class analysis. Transl Issues Psychol Sci. (2018) 4:440. doi: 10.1037/tps0000176

[40] Weller BE Bowen NK Faubert SJ. Latent class analysis: a guide to best practice. J Black Psychol. (2020) 46:287–311. doi: 10.1177/0095798420930932

[41] Celeux G Soromenho G. An entropy criterion for assessing the number of clusters in a mixture model. J Classif. (1996) 13:195–212. doi: 10.1007/BF01246098

[42] Jensen TM Bowen GL King EL. Associations between family maltreatment perpetration and latent profiles of personal and family strengths among active-duty air force members. J Fam Viol. (2022) 37:407–21. doi: 10.1007/s10896-021-00274-5

[43] Muthén LK Muthén BO. Mplus user’s guide. 7th ed. Los Angeles: Muthén & Muthén (2012).

[44] Nylund-Gibson K Grimm RP Masyn KE. Prediction from latent classes: a demonstration of different approaches to include distal outcomes in mixture models. Struct Equ Model Multidiscip J. (2019) 26:967–85. doi: 10.1080/10705511.2019.1590146

[45] Asparouhov T Muthén B. Auxiliary variables in mixture modeling: three-step approaches using Mplus. Struct Equ Modeling. (2014) 21:329–41. doi: 10.1080/10705511.2014.915181

[46] Adjei JK Saewyc EM. Boys are not exempt: sexual exploitation of adolescents in sub-Saharan Africa. Child Abuse Negl. (2017) 65:14–23. doi: 10.1016/j.chiabu.2017.01.001, PMID: 28110108

[47] Cluver L Orkin M Boyes M Gardner F Meinck F. Transactional sex amongst AIDS-orphaned and AIDS-affected adolescents predicted by abuse and extreme poverty. J Acquir Immune Defic Syndr. (2011) 58:336–43. doi: 10.1097/QAI.0b013e31822f0d82, PMID: 21857361

[48] Franchino-Olsen H Martin SL. The associations between gang membership and domestic minor sex trafficking: findings from a nationally representative study. Violence Vict. (2022) 37:479–96. doi: 10.1891/VV-2021-0070, PMID: 35577530 PMC10765547

[49] Hernandez R.. (2021). The commercial sexual exploitation of boys & LGBTQ+ youth: A systematic literature review [master's thesis]. California State University San Marcos. Available online at: https://scholarworks.calstate.edu/downloads/2v23w095f (Accessed April 1, 2024).

[50] Homma Y Nicholson D Saewyc EM. A profile of high school students in rural Canada who exchange sex for substances. Can J Hum Sex. (2012) 21:29–40. 26709343 PMC4690723

[51] McNeal BA Walker JT. Parental effects on the exchange of sex for drugs or money in adolescents. Am J Crim Justice. (2016) 41:710–31. doi: 10.1007/s12103-015-9313-7

[52] Godoy SM Hall WJ Cal T Bath EP Abrams LS Goode RW. Children’s pathways into commercial sexual exploitation in the United States: a systematic review. Trauma Violence Abuse. (2024). doi: 10.1177/15248380241297420, PMID: 39569813

[53] de Vries I Nickerson C Farrell A Wittmer-Wolfe DE Bouché V. Anti-immigration sentiment and public opinion on human trafficking. Crime Law Soc Change. (2019) 72:125–43. doi: 10.1007/s10611-019-09838-5

[54] Heber A. Damsels, monsters, and superheroes: exploring the metanarrative of sex trafficking. Int Rev Victimol. (2024) 30:89–108. doi: 10.1177/02697580231195095

[55] Baird K Connolly J. Recruitment and entrapment pathways of minors into sex trafficking in Canada and the United States: a systematic review. Trauma Violence Abuse. (2023) 24:189–202. doi: 10.1177/15248380211025241, PMID: 34184579 PMC9660274

[56] Haney K LeBeau K Bodner S Czizik A Young ME Hart M. Sex trafficking in the United States: a scoping review. J Evid Based Soc Work. (2020) 17:714–48. doi: 10.1080/26408066.2020.1765934, PMID: 32678726

[57] Reid JA. Entrapment and enmeshment schemes used by sex traffickers. Sex Abus. (2016) 28:491–511. doi: 10.1177/1079063214544334, PMID: 25079777

[58] Sprang G Cole J. Familial sex trafficking of minors: trafficking conditions, clinical presentation, and system involvement. J Fam Violence. (2018) 33:185–95. doi: 10.1007/s10896-018-9950-y

[59] Barron IM Frost C. Men, boys, and LGBTQ: invisible victims of human trafficking In: Walker L Gaviria G Gopal K, editors. Handbook of sex trafficking: Feminist transnational perspectives. Cham: Springer (2018). 73–84.

[60] Phillips J. Black girls and the (im)possibilities of a victim trope: the intersectional failures of legal and advocacy interventions in the commercial sexual exploitation of minors in the United States. UCLA Law Rev. (2015) 62:1642.

[61] Reed SM Kennedy MA Decker MR Cimino AN. Friends, family, and boyfriends: an analysis of relationship pathways into commercial sexual exploitation. Child Abuse Negl. (2019) 90:1–12. doi: 10.1016/j.chiabu.2019.01.016, PMID: 30716650

[62] Allert JL (2021). A mixed-methods descriptive study of domestic minor familial sex trafficking through the lens of justice professionals [dissertation]. Regent University.

[63] Barnert E Kelly M Godoy SM Abrams LS Rasch M Bath EP. Understanding commercially sexually exploited young women's access to, utilization of, and engagement in health care: “work around what I need.”. Womens Health Issues. (2019) 29:315–24. doi: 10.1016/j.whi.2019.02.002, PMID: 30962075 PMC6724548

[64] Godoy SM Abrams L Barnert ES Kelly MA Bath EP. Fierce autonomy: how commercially sexually exploited young women perceive their health and exercise agency in healthcare decision-making. Qual Health Res. (2020) 30:1326–37. doi: 10.1177/1049732320913857 32285750 PMC8262511

[65] Varma S Gillespie S McCracken C Greenbaum VJ. Characteristics of child commercial sexual exploitation and sex trafficking victims presenting for medical care in the United States. Child Abuse Negl. (2015) 44:98–105. doi: 10.1016/j.chiabu.2015.04.004, PMID: 25896617

[66] Le PD Ryan N Rosenstock Y Goldmann E. Health issues associated with commercial sexual exploitation and sex trafficking of children in the United States: a systematic review. Behav Med. (2018) 44:219–33. doi: 10.1080/08964289.2018.1432554, PMID: 30020867

[67] Li X Feigelman S Stanton B. Perceived parental monitoring and health risk behaviors among urban low-income African-American children and adolescents. J Adolesc Health. (2000) 27:43–8. doi: 10.1016/S1054-139X(99)00077-4, PMID: 10867351

[68] Dittus PJ Li J Verlenden JV Wilkins NJ Carman-McClanahan MN Cavalier Y. Parental monitoring and risk behaviors and experiences among high school students-youth risk behavior survey, United States, 2021. MMWR Suppl. (2023) 72:37–44. doi: 10.15585/mmwr.su7201a5, PMID: 37104464 PMC10156152

